# The modulation effect of non-invasive brain stimulation on cognitive function in patients with mild cognitive impairment: a systematic review and meta-analysis of randomized controlled trials

**DOI:** 10.1186/s12868-018-0484-2

**Published:** 2019-01-03

**Authors:** Ying Xu, Zhijie Qiu, Jingfang Zhu, Jiao Liu, Jingsong Wu, Jing Tao, Lidian Chen

**Affiliations:** 1 Rehabilitation Medicine College, Fujian University of Traditional Chinese Medicine, Fuzhou, China; 2 National Rehabilitation Research Center of Traditional Chinese Medicine, Fuzhou, China; 3 Fujian University of Traditional Chinese Medicine, Fuzhou, China

**Keywords:** Non-invasive brain stimulation, Mild cognitive impairment, Cognitive function, Meta-analysis

## Abstract

**Background:**

To prevent and control dementia, many scholars have focused on the transition stage between normal ageing and dementia, mild cognitive impairment (MCI) which is a key interventional target for dementia. Studies have shown that non-invasive brain stimulation (NIBS) is beneficial to improve cognitive function of MCI patients. However, whether NIBS is conducive to the protection of cognitive ability in MCI patients remains unknown due to limited evidence. The aim of the study was to systematically evaluate the modulation effect of NIBS on cognitive function (global cognitive ability and specific domains of cognition) in patients with MCI.

**Results:**

A total of 11 RCTs comprising a total of 367 MCI participants. Meta-analysis showed that NIBS can significantly improve global cognition (n = 271, SMD = 0.94, 95% CI 0.47–1.41, *p* < 0.0001) and verbal fluency (n = 72, MD = 2.03, 95% CI 0.17–3.88, *p* = 0.03). However, there was no significant improvement in other domains of cognition.

**Conclusions:**

NIBS has a positive effect on improving global cognitive function and verbal fluency. At the same time, it has a small positive effect on improving executive function. However, these findings should be interpreted carefully due to the limitations of the study.

**Electronic supplementary material:**

The online version of this article (10.1186/s12868-018-0484-2) contains supplementary material, which is available to authorized users.

## Background

With age increasing, the risk of Alzheimer’s disease (AD) is on the rise [1], however, the treatment of dementia is far from satisfactory [2, 3]. To prevent and control dementia, many scholars have focused on the transition stage between normal ageing and dementia, mild cognitive impairment (MCI) which is a key interventional target for dementia [4]. The main characteristics of MCI are objective memory impairment and other cognitive deficits; however, aspects of daily living are not significantly affected [5]. The incidence of MCI in people over 65 years of age is 10–20%, and more than half of them will progress into dementia within 5 years [6, 7]. Severe cognitive decline will have a huge impact on the daily lives of patients such as independent living ability losing, lower quality of life, and huge economic burden [8]. Thus, effective and timely interventions that aim to improve cognitive function or delay the process of cognitive decline will significantly benefit patients and their families.

Recently, there has been an increased interesting on the use of non-drug therapy to improve the cognitive function of MCI patients [9]. Studies have shown that as a new type of treatment, non-invasive brain stimulation (NIBS) is beneficial to improve cognitive function of MCI patients [10]. NIBS can alter neuronal activity temporarily and affect behavioural performance [11].

The two most commonly used techniques of NIBS are transcranial magnetic stimulation (TMS) and transcranial direct current stimulation (tDCS). TMS modulates cortical activities by delivering strong magnetic pulses to the cortex through the scalp [12, 13]. Different stimulation frequencies can enhance or inhibit cortical excitability in the target cortical region. Unlike TMS, tDCS delivers a continuous week currents (0.5–2.0 mA) to the scalp to modulate neuronal transmembrane potential toward hyperpolarization or depolarization, thereby altering plasticity in the stimulated brain regions [10, 14]. Although they are different in some respects, both tools can induce long-term after effects on cortical excitability and neuroplasticity.

Many studies [15–18] have reported the treatment effect of tDCS and TMS on cognitive outcomes in various populations, such as AD and healthy adults. For example, a study reported that for older adults, compare to the anodal or placebo (sham) tDCS, anodal tDCS not only strengthened episodic memories, but delayed recall is enhanced after 48 h compared with placebo stimulation [19]. Similarly, another study found up-regulation of the dorsolateral prefrontal cortex (DLPFC) led to improvements of everyday memory after 10-Hz TMS in MCI patients [20].

However, beneficial effects of NIBS are not always observed. A study [21] showed that 2 weeks of tDCS did not show significant group differences in the face-name association task. In addition, Boggio et al. [22] found that tDCS over the prefrontal cortex increases high-risk behaviour in older adults. In particular, a study with a crossover design failed to induce positive or negative behavioral effects following either low- or high-frequency TMS in seven patients with vascular MCI [23]. Therefore, the efficacy of tDCS or TMS as a treatment option remains controversial. Specifically, whether tDCS or TMS is conducive to the protection of cognitive ability in MCI patients remains unknown due to limited evidence.

This study was designed to systematically evaluate the effect of tDCS and TMS as an intervention on cognitive function of MCI patients, including global cognitive ability and specific domains of cognition, such as memory, executive function, attention, verbal fluency.

## Methods

### Protocol and registration

The protocol of this study was registered with the International Prospective Register of Systematic Review, PROSPERO, under the identification number CRD42018092620, and can be integrally assessed online (http://www.crd.york.ac.uk/PROSPERO/display_record.php?ID=CRD42018092620).

### Literature search

We searched a total of seven electronic databases, including PubMed, EMBASE (OVID), SinoMed, China National Knowledge Infrastructure (CNKI), Wanfang degree and conference papers database, Chinese Science and Technology Periodical Database (VIP) from its inception to 31 January 2018, as well as the Cochrane Central Register of Controlled Trials (Cochrane Library, 2018, Issue 1). These databases were searched without language restrictions. Relevant keywords related to non-invasive brain stimulation as Medical Subject Heading terms and text words (e.g., ‘noninvasive brain stimulation’, or ‘transcranial direct current stimulation’, or ‘transcranial magnetic stimulation’) were used in combination with words related to mild cognitive impairment.

### Inclusion criteria

The trials selected in the study should met the following inclusion criteria: (1) published or unpublished randomized controlled studies; (2) participants diagnosed with MCI based on any diagnostic criteria, such as Petersen criteria, 2004 MCI Key Symposium criteria or other standards and consensus; participants with vascular cognitive impairment or other neurological disorders resulting from AD, dementia or Parkinson’s disease were excluded; (3) the intervention of the experimental group was NIBS technique, regardless of the type; (4) the control group received basic intervention, sham stimulation, medication or other interventions; (5) outcomes included global cognitive ability and specific domain of cognition, which was measured by neuropsychological tests or other objective measurements. Studies without available data were excluded.

### Study identification and data extraction

Eliminate duplicate records with the reference management software (Note Express V.2.0). In the literature screening, the title and abstracts were read first to eliminate irrelevant studies. The studies that potentially met the inclusion criteria were independently screened, extracted, and cross-checked by two reviewers. Disagreements were resolved by discussion with a third reviewer. Extract the following information from eligible studies: study design, sample size, participants’ characteristics, methodological information of research quality, experimental and control intervention, the duration, frequency, intensity and type of NIBS, outcomes, the time of follow-up, and adverse events.

### Assessment of risk of bias in individual studies

Two reviewers independently evaluated the scientific quality of the study according to the JADAD scoring manual [24]. Evaluation content included description of random sequence generation, description of the double-blind procedure, and description of withdrawals and dropouts. The total score was 5 points, where a score of 1–2 points was associated with low quality and a score of 3–5 points was deemed as high quality.

### Data analysis

A meta-analysis of outcomes for each study was performed using Review Manager 5.3 software and a two-sided *p* < 0.05 was considered statistically significant. Data were summarized using relative risk with 95% CI for binary outcomes. The measurement data used the MD or standardized MD and the 95% CI as the effect amount. The meta-analysis used the I^2^ test to observe the degree of statistical heterogeneity between studies. When I^2^ ≤ 50%, a fixed-effect model was used, while a random-effects model was used when I^2^ > 50%. A parallel sensitivity analysis was used to find the source of heterogeneity. Studies with different interventions were divided into subgroups for subgroup analysis according to different factors, such as design options, treatment duration, and specific interventions. For studies with significant clinical and methodological heterogeneity, the outcome meta-analysis was not performed, and only general statistical descriptions were performed. Statistical heterogeneity among the included studies was assessed using a χ^2^ test and Higgins I^2^ value, with I^2^ > 75% suggesting high statistical heterogeneity [25].

## Results

### Study identification

According to the original search strategy, 510 studies were retrieved, and 72 repetitive studies were excluded. After reading titles and abstracts, two independent reviewers excluded 416 articles that did not meet the inclusion criteria. A total of 22 studies were further evaluated, excluding 1 repeated publication, while 2 did not meet the MCI diagnostic criteria, 4 had unacceptable cognitive outcomes, and 4 were non-RCT studies. A total of 11 studies [20, 26–35] were included in the meta-analysis finally.

**Fig. 1:**
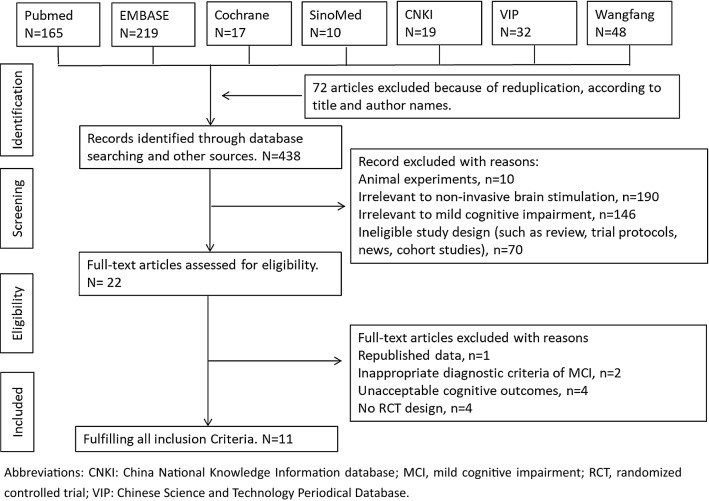
Flow diagram for searching and selection of the included studies

The detailed screening flow used to find eligible studies is presented in Fig. 1.

### Characteristics of included studies

This review included 11 RCT studies involving 367 MCI participants (175 males and 192 females, average age 66.52 years) and Table 1 shows the characteristics of each study. Six studies [28–33] were conducted in China, two [26] in Italy, one [27] in Brazil, one [34] in South Korea and one [35] in the US. All studies reported clear diagnostic, inclusion, and exclusion criteria.

**Table 1:** Characteristics of included studies in this systematic review

Author, year	Mean age	Participants (M/F)	Intervention	Stimulation site	Frequency, duration and intensity	Outcomes
Han, 2013	66.59	40 (14/26)	T: active rTMS C: sham rTMS	Left/right DLPFC	20 Hz, 80% RMT, 30 min/day, 5 days/week, 8 weeks	Global cognitive function/MoCA; Linguistic function/VFT; Executive function/WCST; Attention/TMT-A, DSST
Zhang, 2014	65.75	50 (25/25)	T: active rTMS C: oral Piracetam	Bilateral frontal area	5 Hz, 100% RMT, 800 pulses/day, 6 days/week, 16 weeks	Global cognitive function/MoCA; ERP
Yang, 2014	66.00	33 (15/18)	T: active rTMS C: sham rTMS	Bilateral DLPFC	20 Hz, 80% RMT, 30 min/day, 5 days/week, 8 weeks	Global cognitive function/MMSE; ERP
Rosa, 2015	69.05	20 (11/9)	T: anode tDCS + PT C: sham tDCS + PT	Left DLPFC	2 mA, 25 min/day, 5 days/week, 2 weeks	Global cognitive function/MMSE, PD-CRS; Memory/PAL; Attention/TMT; Executive function/Semantic fluency
Sun, 2015	64.40	80 (43/37)	T: active rTMS + cognitive training C: cognitive training	Left DLPFC and left PC	15 Hz, 80%-110%RMT, 30 min/day, 6 days/week, 8 weeks	Global cognitive function/MoCA; ERP
Hellen, 2015	65.16	34 (12/22)	T: active rTMS C: sham rTMS	Left DLPFC	10 Hz, 110% RMT, 2000 pulses/session, 1 session/day, 10 days	Memory/RBMT, WMS, RAVLT, WAIS-III; Executive function/TMT-B, VFT
Kyongsik, 2016	73.94	16 (5/11)	T: anode tDCS C: sham tDCS	Anodal: left DLPFC; Cathode: right DLPFC	2 mA, 30 min/day, 3 days/week, 3 weeks	Memory/MMQ, HVLT; Visuospatial function/RCFT; Linguistic function/BNT; PET
Long, 2016	66.95	30 (14/16)	T: active rTMS C: sham rTMS	Left DLPFC	15 Hz, 90% RMT, once a day, 10 days	Global cognitive function/MoCA; Memory/CMS
Wu, 2017	67.61	41 (21/20)	T: active rTMS + oral paroxetine C: oral paroxetine	Left DLPFC	20 Hz, 80% RMT, 15 min/day, 5 days/week, 4 weeks	Global cognitive function/MMSE; ERP
Giacomo, 2017	70.00	14 (7/7)	T: active rTMS C: sham rTMS	PC	20 Hz, 100% RMT, 20 min/day, 5 days/week, 6 weeks	Global cognitive function/MMSE, Memory/RAVLT; Executive functions/FAB; Attention/DSST
Prasad, 2018	65.60	9 (8/1)	T: active rTMS C: sham rTMS	Left DLPFC	10 Hz, 120% RMT, 45 min/day, 5 days/week, 8 weeks	Apathy/AES-C; Global cognitive function/MMSE; Executive functions/TMT A&B; Functional status/IADL; Impression/CGI

BNT, Boston Naming Test; CMS, Clinical Memory Scale; CGI, Clinical Global Impression; DLPFC, dorsolateral prefrontal cortex; DSST, Digit Symbol Substitution Test; ERP, Event-related Potentials; FAB, Frontal Assessment Battery; HVLT, Hopkins Verbal Learning Test; IADL, Instrumental Activities of Daily Living; MMSE, Mini-mental State Examination; MMQ, Multifactorial Memory Questionnaire; MoCA, Montreal Cognitive Assessment; PAL, Paired Associated Learning; PC, Precuneus; PD-CRS, Parkinson’s Disease Cognitive Rating Scale; PET, Positron Emission Tomography; PT, Physical Therapy; RAVLT, Rey Auditory Verbal Learning Test; RBMT, Rivermead Behavioral Memory Test; RCFT, Rey Complex Figure Test; RMT, Resting Motor Threshold; rTMS, repetitive Transcranial Magnetic Stimulation; TMT, Trial Making Tests; tDCS, transcranial Direct Current Stimulation; VFT, Verbal Fluency Test; WAIS-III, Wechsler Adult Intelligence Scale III; WCST, Wisconsin Card Sorting Test; WMS, Wechsler Memory Scale

The types of NIBS included tDCS [26, 27] and TMS [20, 28–35]. For tDCS, the frequency varied from three to five sessions weekly and 25–30 min per session. The duration of the intervention lasted 2–3 weeks. The stimulation site was in the dorsolateral prefrontal cortex (DLPFC), and the intensity was 2 mA. For TMS, the frequency varied from five to six sessions weekly and 15–45 min per session. The duration of the intervention lasted 2–16 weeks. The stimulation site was in the DLPFC, bilateral frontal area, and precuneus, with an intensity from 80 to 120% resting motor threshold (RMT). Of these 11 studies, 3 were followed up from 1 to 3 months [20, 26, 33]. Two studies compared TMS with drug therapy [29, 33]. Two studies combined TMS/tDCS with physical therapy (PT) or cognitive training to compare the effects of combination therapy with PT or cognitive training alone [26, 31]. Other studies performed comparisons between true and shame-stimuli. There was a wide variety of cognitive measurement tools used in these studies, including Mini-mental state examination (MMSE), Montreal Cognitive Assessment (MoCA), Parkinson’s Disease Cognitive Rating Scale (PD-CRS), Wechsler Memory Scale (WMS), Trial Making Tests-A and B (TMT-A&B), and verbal fluency test (VFT), with different tools applied to evaluate the same cognitive domain within a study or among studies.

### Risk of bias of included studies

The risks of bias for all studies are shown in Table 2. In these studies, 3 studies described the use of a random number table and Matlab software to generate random sequences [30, 33, 34]. One study used the covariate adaptive randomization method for random assignment [26]. The rest of the studies mentioned random grouping but did not describe the generation method of random sequences, and there was a potential risk of high selection bias. Five studies used a double-blind design for blindness to subjects and assessors [20, 26, 30, 34, 35]; therefore, their risk of detection bias was judged as low. A single-blind design was used in one other study [33]. However, none of the other studies mentioned blindness, and thus have a potentially high risk of implementation bias and high detection bias. Four studies reported shedding and described the data processing method [20, 26, 30, 34, 35]; therefore, the risk of loss bias was deemed as low. Overall, most of the included studies were judged to have a high risk of bias with low methodological quality.

**Table 2:** Risk of bias summary: review authors’ judgements about each risk of bias item for each included study

Author, year	Randomization sequence generation	Blinding method	Withdrawals and dropouts	Jadad score
Han, 2013	Not described in detail	Not mentioned	2 Drop out of family reason	2
Zhang, 2014	Not described in detail	Not mentioned	Not mentioned	1
Yang, 2014	Random number table	Double-blind manner	Not mentioned	4
Rosa, 2015	Covariate adaptive randomization method	Double-blind manner	Not mentioned	4
Sun, 2015	Not described in detail	Not mentioned	Not mentioned	1
Hellen, 2015	Not described in detail	Double-blind manner	2 Drop out of personal reason	4
Kyongsik, 2016	Random number generator from the Matlab software	Double-blind manner	Not mentioned	4
Long, 2016	Not described in detail	Not mentioned	Not mentioned	1
Wu, 2017	Random number table	Single-blind manner	7 Drop out of a change in condition or other reasons	3
Giacomo, 2017	Not described in detail	Not mentioned	Not mentioned	3
Prasad, 2018	Not described in detail	Double-blind manner	1 Did not tolerate the treatment	4

CNKI: China National Knowledge Information database; MCI, mild cognitive impairment; RCT, randomized controlled trial; VIP: Chinese Science and Technology Periodical Database

### Effect of interventions

#### Global cognitive function

**Fig. 2:**
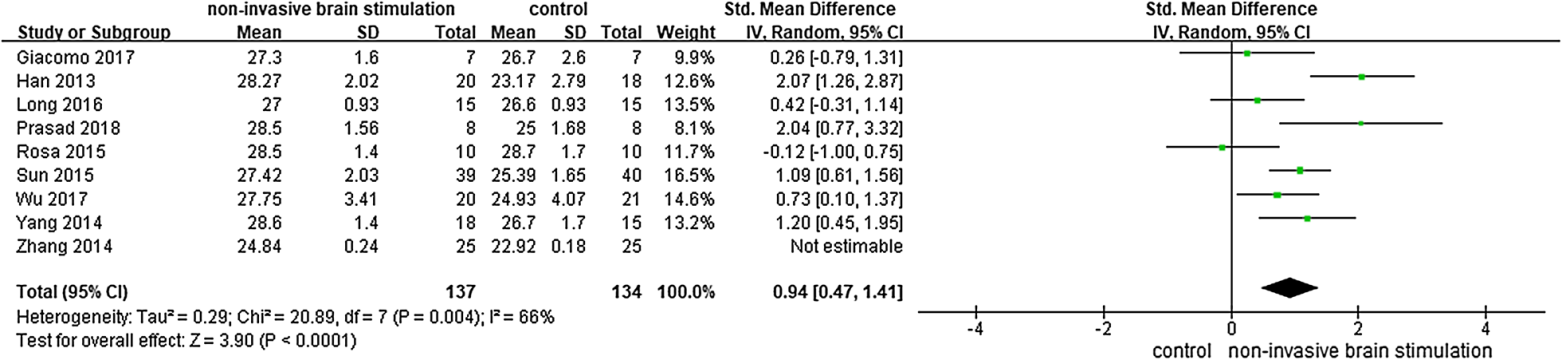
NIBS versus other intervention: global cognitive ability

Nine studies [26, 28–34] reported the effects of NIBS on global cognitive ability in participants with MCI by using MMSE, MoCA and Parkinson’s Disease Cognitive Rating Scale (PD-CRS). The results showed that NIBS had a significant effect on improving global cognitive ability among participants with MCI, as demonstrated by significantly increased MMSE scores and MoCA scores (n = 301, SMD = 1.82, 95% CI 0.86–2.78, *p* < 0.0002, I^2^ = 91%, the random-effect model; Fig. 2). One study [26] reported that tDCS participants had higher PD-CRS scores than those in the control group, the difference was significant (*p* = 0.041).

#### Memory

**Fig. 3:**
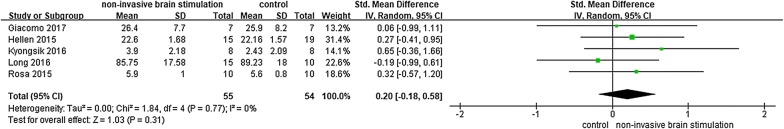
NIBS versus other intervention: memory

Five studies [20, 26, 27, 32, 34] involving 109 participants reported the effects of NIBS on memory ability by using the Rivermead Behavioural Memory Test (RBMT), Rey Auditory-Verbal Learning Test (RAVLT), Digit Symbol Substitution Test (DSST), Multifactorial Memory Questionnaire (MMQ), and Clinical memory scale (CMS). Three of the studies compared the effects of TMS stimulation and sham TMS stimulation. The other two articles compare the difference between anode tDCS stimulation and PT/sham stimulation. The results of meta-analysis showed no significant difference between the NIBS group and control groups (n = 109, SMD = 0.20, 95% CI − 0.18 to 0.58, *p* = 0.31, I^2^ = 0%, the random-effect model; Fig. 3).

#### Executive function

**Fig. 4:**
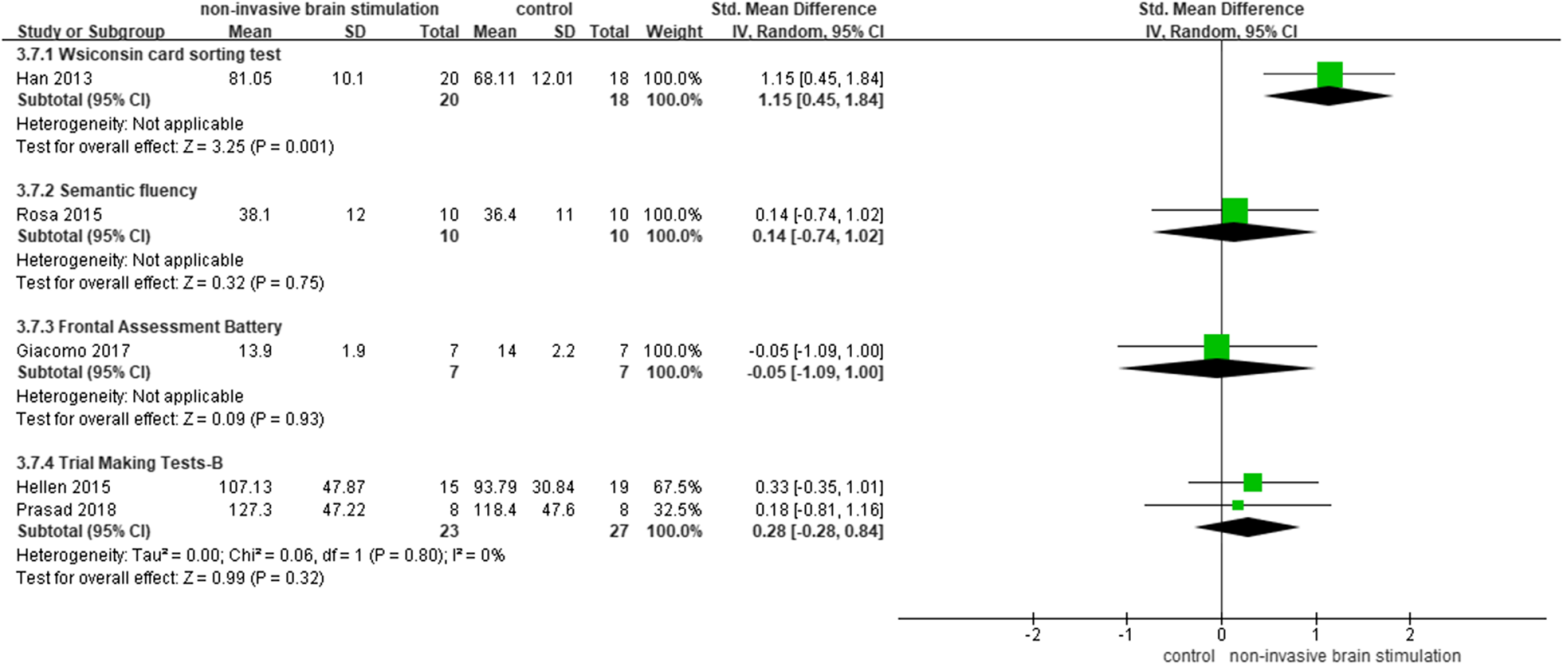
NIBS versus other intervention: executive ability

The effects of NIBS on executive function were evaluated in five studies [20, 26, 28, 34, 35] using the Wisconsin card sorting test (WCST), Semantic fluency and Frontal Assessment Battery (FAB), and TMT part B (ms). Due to the use of these different tools, we performed a subgroup analysis. One study showed that TMS significantly improved executive ability by increasing WCST scores (SMD = 1.15, 95% CI 0.45–1.84, *p* = 0.001), while another reported no significant changes (Fig. 4).

#### Attention

**Fig. 5:**
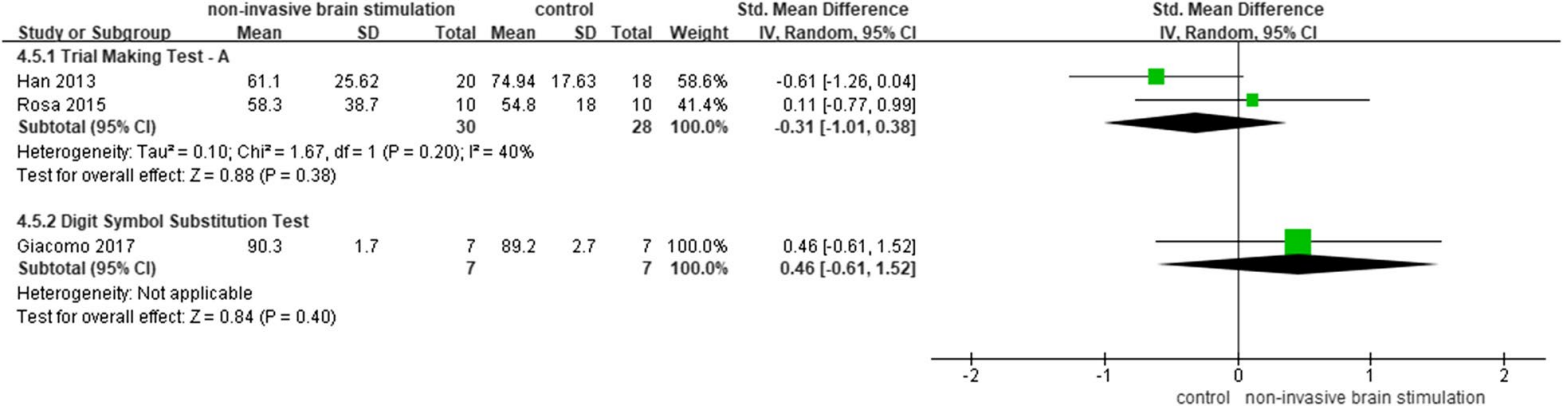
NIBS versus other intervention: attention

Three studies [26, 28, 34] reported the effects of NIBS on attention ability by using TMT part A (ms) and Digit Symbol Substitution Test (DSST). The results of meta-analysis showed no significant difference between the NIBS group and control groups on reaction time of TMT part A (n = 58, SMD = − 0.31, 95% CI − 1.01 to 0.38, *p* = 0.38, I^2^ = 40%, the random-effect model). Regarding the DSST score, NIBS had no significant effect on improving attention ability (n = 14, MD = 0.46, 95% CI − 0.61 to 1.52, *p* = 0.40; Fig. 5).

#### Verbal fluency

**Fig. 6:**
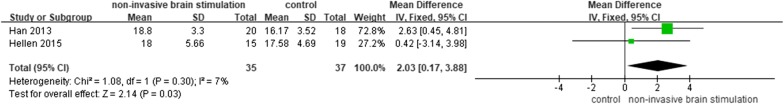
NIBS versus other intervention: verbal fluency

Two studies [20, 28] reported the effects of NIBS on verbal fluency ability. The results of meta-analysis showed a significant difference between NIBS and control group (n = 72, MD = 2.03, 95% CI 0.17–3.88, *p* = 0.03, I^2^ = 7%, the fixed-effect model; Fig. 6).

#### Adverse effects

**Fig. 7:**
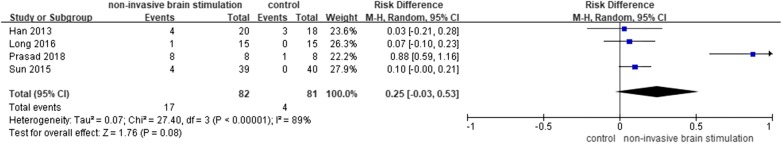
NIBS versus other intervention: adverse effects

Adverse effects were reported in 4 studies [28, 31, 32, 35], where the main symptoms were dizziness, pain, and facial twitching. The results of meta-analysis showed no significant difference between the NIBS group and control groups on adverse event rate (RD = 0.25, 95% CI − 0.03 to 0.53, *p* = 0.08, I^2^ = 89%, the random-effect model; Fig. 7). Notably, two participants experienced severe pain during TMS stimulation, while one participant quit treatment.

## Discussion

Epidemiological studies show that 10–15% patients with MCI will transform to dementia [36]. It is very important to provide timely interventions for patients with cognitive impairment in this reversible phase. NIBS has become a potentially useful tool. In particular, NIBS can directly affect the memory mechanisms of young, elderly, and neurotic dysfunction patients, including working memory, episodic memory, and contact memory [37, 38]. However, there is no consistent conclusion regarding whether NIBS can improve the cognitive function of MCI patients.

Eleven studies involving 367 subjects were included in this review. All studies were designed to compare tDCS or TMS with a lack of specific stimuli. The results of the meta-analysis showed that NIBS was beneficial to improve the cognitive function of MCI patients. In terms of global cognitive function, NIBS significantly improved the global cognitive function in MCI patients. Within a specific cognitive domain, NIBS has a slightly significant benefit on executive function and a potentially positive impact on verbal fluency ability. Nevertheless, as verbal fluency was measured only in two studies, the results of this measure should be taken with caution.

With functional magnetic resonance imaging (fMRI), multiple resting state networks such as default mode network, attention network and sensorimotor network have been affected in patients with MCI [39]. Meinzer et al. conducted a double-blind, cross-control study in which brain changes were recorded using task-related and resting fMRI during tDCS stimulation. fMRI data suggest that the low accuracy of semantic flow tests in MCI patients may be related to hyperactivity of bilateral prefrontal area. Anodic-tDCS significantly improved the accuracy of semantic fluency tests in MCI patients, reduced task-related prefrontal hyperactivity and facilitated the normalization of abnormal network structure in resting-state fMRI [40]. Another critical one concerns with modulation of neurotransmitter levels [41, 42]. TMS may affect the regulation of cortical neuronal activity by altering the dynamics of excitation/inhibition of neurotransmitter systems, such as GABA and glutamate [42].

Previous studies and reviews have summarized the main advantages of tDCS and TMS [10, 43]: first, these methods are ideal for exploring brain plasticity throughout the life cycle. Second, they can regulate neurons bidirectionally, not only inhibiting the excitability of neurons but also enhancing the excitability of neurons. Third, these techniques can apply the stimulus only to the position we want without affecting other parts, so that we can treat the disease while avoiding side effects. This is beyond the ability of pharmacology or complementary therapy. In particular, the application of NIBS to dysfunctional neural networks could significantly enhance learning- and memory-related effects. Moreover, repeated use of NIBS for stimulation could make the effect longer lasting.

In terms of safety, there were several mild adverse events in the NIBS group and control group, such as temporary dizziness, pain, and facial twitching. However, these events could be recovered without special treatment. In addition, there was no significant difference in the incidence of adverse events between the two groups. However, it should be noted that there were 2 serious adverse events in the intervention group, of which one participant had severe pain during the treatment, where the pain was relieved after the stimulus was stopped, while the other patient quit treatment due to pain. Although the safety of NIBS has been recognized by most people currently, the individual safety should be monitored, and personal thresholds should be evaluated to determine appropriate parameters for each patient undergoing NIBS treatment [44–46].

This review only included randomized controlled trials, which implied that the included studies had rigorous research design. Regarding the participants, we more force on the patients with MCI. In order to reduce potential confusion and make the generalization of the findings more pertinent, we excluded participants with secondary cognitive impairment (e.g., vascular dementia) or severe cognitive impairment (e.g., AD). All these methods helped to support the NIBS-related causal hypothesis.

There were several limitations in the systematic review. (1) The stimulation parameters used in each study (including stimulation intensity, stimulation rate, stimulation site, and duration) were quite different, and the optimal parameters for NIBS treatment could not be determined. (2) Only three studies were followed up for 1–3 months, suggesting that the effect of NIBS could be maintained for a long time; however, the conclusion should be verified by larger and longer follow-up studies. (3) The control methods adopted in the study were different. Due to the small number of included studies, the systematic review included the results of all the studies together for analysis but did not analyse them separately according to the methodological differences. For example, there are studies that only used drugs as controls, and thus, the placebo effect could not be avoided, which may exaggerate the efficacy of NIBS. Moreover, some studies did not describe whether the subjects were combined with the basic treatment. Therefore, it is unclear whether there was a synergistic effect between the treatments. (4) The overall quality of the research included in this systematic review was not high. Most of the studies did not specifically describe the method of random allocation and did not perform allocation concealment, which may generate selection bias. (5) Eight studies used shame stimuli, but did not test whether the blindness was successful, which may increase measurement bias.

### Clinical implications and recommendations for future studies

Meta-analysis showed that in patients with MCI, NIBS seems to improve the overall cognitive function, verbal fluency, and executive function, which suggests that NIBS may be a potential intervention for patients with MCI. Because the quality of the studies is not high, the conclusions should be treated with caution. We also believe that NIBS may offer an exciting novel treatment option in patients with MCI.

Future studies in this field should explore appropriate NIBS treatment parameters (e.g., stimulus type, stimulus intensity, stimulation frequency, stimulation site) for patients with MCI. For example, Ahmed et al. [47] conducted a TMS intervention trial on AD, suggesting that high-frequency stimulation is superior to low-frequency stimulation. Moreover, a more sensitive and objective measurement tool should be used to assess the overall cognitive ability and specific domains of cognition. As memory loss is a major manifestation of MCI, recent studies have concluded that delayed memory and semantic memory tests can better predict whether MCI will progress to AD compared to other memory tests [48].

There are different types of MCI, such as amnestic MCI and non-amnestic MCI as well as single domain impairment or multiple domain impairment [49]. Whether the response of different types of MCI to NIBS treatment is different remains to be discussed. In addition, since the adverse events reported in these studies are mainly transient dizziness and headaches, we believe that the setting of NIBS parameters in future studies should be based on safety guidelines [50–52]. At the same time, follow-up studies are needed to assess the long-term risks and benefits of NIBS treatment. In addition, in order to better assess the quality of the research, the author should follow the CONSORT guidelines [53] when reporting research.

## Conclusion

NIBS may benefit the improvement of global cognitive function and verbal fluency in patients with MCI, while it has a slight positive impact on executive function. However, considering the different types of NIBS, and the discrepancies in intensity, frequency, locus, duration of stimulation, as well as the limited number of studies and the small sample size, these findings must be carefully explained. More sample size trials are needed, with more rigorously randomized controlled trial designed and standardized training programmes to draw specific and accurate conclusions.

## Additional file

**Additional file 1** Search strategy.

## Abbreviations

AD: Alzheimer’s disease
BNT: Boston Naming Test
CMS: Clinical Memory Scale
CNKI: China National Knowledge Infrastructure
CGI: Clinical Global Impression
DLPFC: dorsolateral prefrontal cortex
DSM-IV: diagnostic and statistical manual
DSST: Digit Symbol Substitution Test
ERP: event-related potentials
FAB: frontal assessment battery
fMRI: functional magnetic resonance imaging
HVLT: Hopkins Verbal Learning Test
IADL: instrumental activities of daily living
MCI: mild cognitive impairment
MMSE: mini-mental state examination
MMQ: Multifactorial Memory Questionnaire
MoCA: Montreal Cognitive Assessment
NIBS: non-invasive brain stimulation
PAL: paired associated learning
PC: Precuneus
PD-CRS: Parkinson’s Disease Cognitive Rating Scale
PET: positron emission tomography
PT: physical therapy
RAVLT: Rey Auditory Verbal Learning Test
RBMT: Rivermead Behavioral Memory Test
RCFT: Rey Complex Figure Test
RCT: randomized controlled studies
RMT: resting motor threshold
TMS: transcranial magnetic stimulation
TMT: Trial Making Tests
tDCS: transcranial direct current stimulation
VFT: Verbal Fluency Test
VIP: Chinese Science and Technology Periodical Database
WAIS-III: Wechsler Adult Intelligence Scale III
WCST: Wisconsin Card Sorting Test
WMS: Wechsler Memory Scale
