# Supplementary material for: The modulation effect of non-invasive brain stimulation on cognitive function in patients with mild cognitive impairment: a systematic review and meta-analysis of randomized controlled trials
Source: BMC Neurosci. 2019 Jan 3;20:2. doi: 10.1186/s12868-018-0484-2 (PMC6317253; doi:10.1186/s12868-018-0484-2)
Supplement: Supplementary file 1 — Additional file 1 Search strategy. [file 12868_2018_484_MOESM1_ESM.docx]

Additional file 1: The detail of search strategy

PubMed Cochrane

#1: non-invasive brain stimulation [All fields]

#2: noninvasive brain stimulation [All fields]

#3: transcranial direct current stimulation [MeSH Terms]

#4: transcranial magnetic stimulation [MeSH Terms]

#5: (#1 OR #2 OR #3 OR #4)

#6: mild cognitive impairment [MeSH Terms]

#7: MCI [All fields]

#8: mild neurocognitive disorder [All fields]

#9: (#6 OR #7 OR #8)

#10: #5 and #9

Embase

#1: (non-invasive brain stimulation or noninvasive brain stimulation or transcranial direct current stimulation or transcranial magnetic stimulation).mp. [mp=title, abstract, subject headings, heading word, drug trade name, original title, device manufacturer, drug manufacturer, device trade name, keyword]

#2: (mild cognitive impairment or MCI or mild neurocognitive disorder).mp. [mp=title, abstract, subject headings, heading word, drug trade name, original title, device manufacturer, drug manufacturer, device trade name, keyword]

#3: #1 and #2

SinoMed

检索式为：("轻度认知障碍"[全字段:智能]) AND "非侵入性脑刺激"[全字段:智能]

("轻度认知障碍"[全字段:智能]) AND "经颅直流电刺激"[全字段:智能]

("轻度认知障碍"[全字段:智能]) AND "经颅磁刺激"[全字段:智能]

维普

检索式为：主题=轻度认知障碍 与主题=非侵入性脑刺激 与主题=经颅直流电刺激 与 主题段=经颅磁刺激 与 范围=全部期刊

万方

检索式为：主题: ("轻度认知障碍") * 主题: ("经颅磁刺激") * 主题:(经颅直流电刺激) Date:-2018

CNKI

中国学术期刊网络出版总库

检索式为：(主题=轻度认知障碍) AND (主题=非侵入性脑刺激)

(主题=轻度认知障碍) AND (主题=经颅直流电刺激)

(主题=轻度认知障碍) AND (主题=经颅磁刺激)
